# Discovery of glycocholic acid and taurochenodeoxycholic acid as phenotypic biomarkers in cholangiocarcinoma

**DOI:** 10.1038/s41598-018-29445-z

**Published:** 2018-07-23

**Authors:** Won-Suk Song, Hae-Min Park, Jung Min Ha, Sung Gyu Shin, Han-Gyu Park, Joonwon Kim, Tianzi Zhang, Da-Hee Ahn, Sung-Min Kim, Yung-Hun Yang, Jae Hyun Jeong, Ashleigh B. Theberge, Byung-Gee Kim, Jong Kyun Lee, Yun-Gon Kim

**Affiliations:** 1 School of Chemical and Biological Engineering, Seoul National University, Seoul, 08826 Korea; 2 Departments of Chemistry and Molecular Biosciences, Northwestern University, Evanston, Illinois 60208 United States; 3 Division of Gastroenterology, Department of Medicine, Samsung Medical Center, Sungkyunkwan University, Seoul, 0635 Korea; 4 Department of Chemical Engineering, Soongsil University, Seoul, 06978 Korea; 5 Department of Biological Engineering, Konkuk University, Seoul, 05029 Korea; 6 Department of Chemistry, University of Washington, Box 351700, Seattle, WA 98195 United States

## Abstract

Although several biomarkers can be used to distinguish cholangiocarcinoma (CCA) from healthy controls, differentiating the disease from benign biliary disease (BBD) or pancreatic cancer (PC) is a challenge. CCA biomarkers are associated with low specificity or have not been validated in relation to the biological effects of CCA. In this study, we quantitatively analyzed 15 biliary bile acids in CCA (n = 30), BBD (n = 57) and PC (n = 17) patients and discovered glycocholic acid (GCA) and taurochenodeoxycholic acid (TCDCA) as specific CCA biomarkers. Firstly, we showed that the average concentration of total biliary bile acids in CCA patients was quantitatively less than in other patient groups. In addition, the average composition ratio of primary bile acids and conjugated bile acids in CCA patients was the highest in all patient groups. The average composition ratio of GCA (35.6%) in CCA patients was significantly higher than in other patient groups. Conversely, the average composition ratio of TCDCA (13.8%) in CCA patients was significantly lower in all patient groups. To verify the biological effects of GCA and TCDCA, we analyzed the gene expression of bile acid receptors associated with the development of CCA in a CCA cell line. The gene expression of transmembrane G protein coupled receptor (TGR5) and sphingosine 1-phosphate receptor 2 (S1PR2) in CCA cells treated with GCA was 8.6-fold and 3.4-fold higher compared with control (untreated with bile acids), respectively. Gene expression of TGR5 and S1PR2 in TCDCA-treated cells was not significantly different from the control. Taken together, our study identified GCA and TCDCA as phenotype-specific biomarkers for CCA.

## Introduction

Cholangiocarcinoma (CCA) is a malignant tumor of the bile duct epithelium^1^. Because of its deep-seated location and weak initial symptoms, CCA is difficult to diagnose and is therefore associated with a high mortality rate^2^. According to a recent statistical survey, the median overall survival of CCA is 25 to 28 months, and the 5-year survival rate is close to 25%^3^. Five-year overall survival in patients at stages III and IV is only 10% and 0%, respectively^4^. In the United States, more than 7,000 CCA patients died each year since 2013, and the mortality rate has also increased by 36% from 1999 to 2014^3^. Therefore, it is considered very important to discover biomarkers for accurate prognosis and precise diagnosis of CCA.

Carbohydrate antigen 19-9 (CA19-9) and carcinoembryonic antigen (CEA) are the most commonly used CCA biomarkers in clinical practice^5^. However, it is difficult to distinguish CCA from benign biliary disease (BBD) and pancreatic cancer (PC) using those biomarkers^6^. Several specific CCA biomarker studies have been performed to overcome these limitations^7^. MicroRNA-21 (miR-21) has been studied as a representative transcriptional biomarker of CCA^8^. Although it has a high sensitivity and is associated with CCA tumor growth, it is tissue-specific and is up-regulated in various diseases^9,10^. Proteomic biomarkers that distinguish CCA specifically include insulin-like growth factor-1 and elastase^11^. However, these biomarkers are not specific for other diseases or most of them have yet to be investigated in the pathogenesis of CCA. Heretofore, studies that are investigating in CCA metabolomic biomarkers are few compared with transcriptomic or proteomic biomarkers. Metabolic biomarkers are more powerful because they accurately represent molecular phenotypes than other markers^12^. In this study, we discovered a specific CCA metabolic biomarker that is expressed abundantly in biles of patients diagnosed with CCA compared with patients suffering from BBD and PC.

Unlike other biofluids such as serum or urine, bile is located close to the biliary tumor, increasing the probability of detection of CCA-specific biomarkers^13^. Additionally, specific bile acids act as signaling molecules associated with CCA pathogenesis and have location-specific properties^14^. Therefore, several studies have recently analyzed bile acids for the discovery of a potential biomarker of CCA^15–17^. The proportion of deoxycholic acid (DCA) in CCA patients was reported to be lower than in patients with biliary tract stones or normal subjects^16^. Other research groups reported that the proportion of cholic acid (CA) and chenodeoxycholic acid (CDCA) was higher in patients with CCA than in BBD or hepatocellular carcinoma (HCC)^18^. Patients with CCA patients carry higher levels of taurine- and glycine-conjugated bile acids compared with patients diagnosed with BBD. However, these studies analyzed fewer samples or species of bile acids and failed to interpret the role of specific bile acid biomarkers in the development of cholangiocarinoma.

In this study, biliary bile acids were quantitatively analyzed by liquid chromatography tandem mass spectrometry (LC-MS/MS) in BBD (n = 57), PC (n = 17) and CCA patients (n = 30) to identify biomarkers with diagnostic potential. As biomarker candidates, we selected glycocholic acid (GCA) and taurochenodeoxycholic acid (TCDCA) with statistically significant differences in bile acid composition among disease groups. Furthermore, in order to verify the biological phenotype associated with the selected CCA bile acid biomarker, we compared the expression level of farnesoid X receptor (FXR), transmembrane G protein-coupled receptor (TGR5) and sphingosine 1-phosphate receptor 2 (S1PR2) genes by qRT-PCR (Quantitative real-time reverse-transcriptase polymerase chain reaction) analysis after the treatment of CCA cell lines with GCA, TCDCA and control, respectively. Based on these studies, we identified phenotypic biomarkers for CCA-specific diagnosis.

## Results and Discussion

### Establishment of a quantitative method for human major bile acids based on LC-MS/MS

There are 15 types of major bile acids in human bile (Supplementary Fig. S1)^19^. Primary bile acids (e.g., CA, CDCA) are synthesized from cholesterol in the liver and conjugated with glycine or taurine. Upon entry into the intestine along the bile duct, they are deconjugated or transformed into secondary bile acids (e.g., DCA, LCA, and UDCA) by gut microbiota. As they pass through the intestines, they are mostly reabsorbed into the hepatic circulation. In this study, we quantified 15 major bile acids in patient groups via LC-MS/MS-based targeted metabolomics approach.

**Figure 1:**
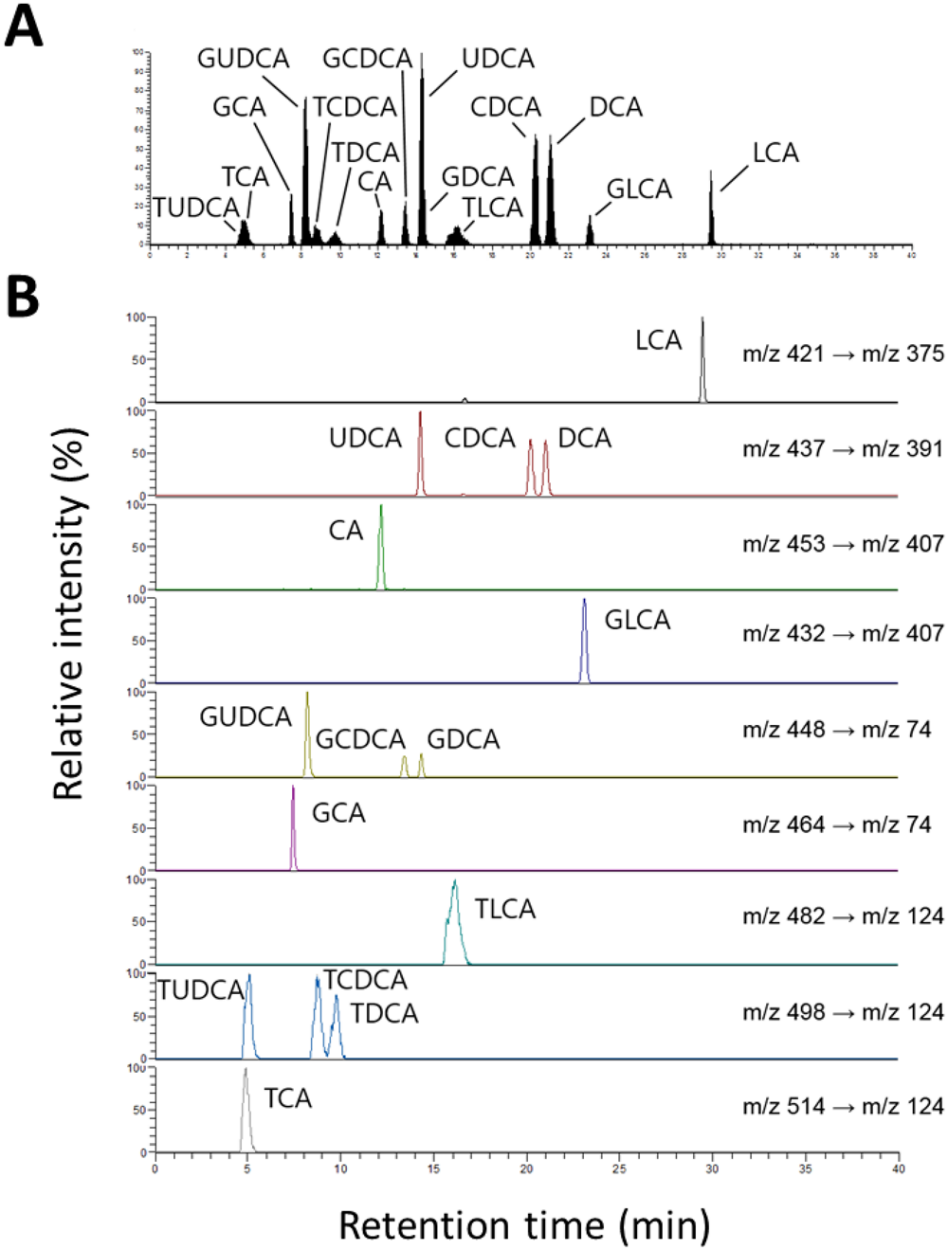
(**A**) Total ion chromatogram separated 15 species of bile acids and (**B**) SRM chromatograms and conditions of bile acids.

Development of an accurate LC-MS/MS quantitative method is essential to quantify biliary bile acids in patients with BBD, PC, and CCA^20^. First, we established the selected reaction monitoring (SRM) conditions for standard compounds of each bile acid and developed a chromatographic method to separate these bile acids. In the precursor ion scan mode, glycine-conjugated bile acids and taurine-conjugated bile acids were detected at molecular mass-to-charge ratio corresponding to [M-H]^−^ ions, respectively. However, the precursor ions of unconjugated bile acids additionally had a molecular mass corresponding to formic acid adduct ion (i.e. [M-H + 46]^−^) (Supplementary Fig. S2)^21^. MS/MS analysis of all bile acids revealed that the neutral loss of a formic acid (46 Da) occurred only from the unconjugated bile acids (Supplementary Fig. S3). With respect to conjugated bile acids, their product ions were observed at *m/z* values of 74 (glycine) and 124 (taurine) from glycine-conjugated and taurine-conjugated compounds, respectively (Supplementary Fig. S3). The quantitative SRM conditions of each bile acid were set using the precursor and fragment ion mass information. Based on these SRM conditions, a 40-min reverse phase LC method was developed to separate all the 15 bile acids (Fig. 1). Quantitative standard curves for 15 bile acid compounds were established to analyze biliary bile acid levels in each patient (Supplementary Fig. S4).

### Comparison of bile acid composition in patients with benign biliary disease, pancreatic cancer, and cholangiocarcinoma

**Figure 2:**
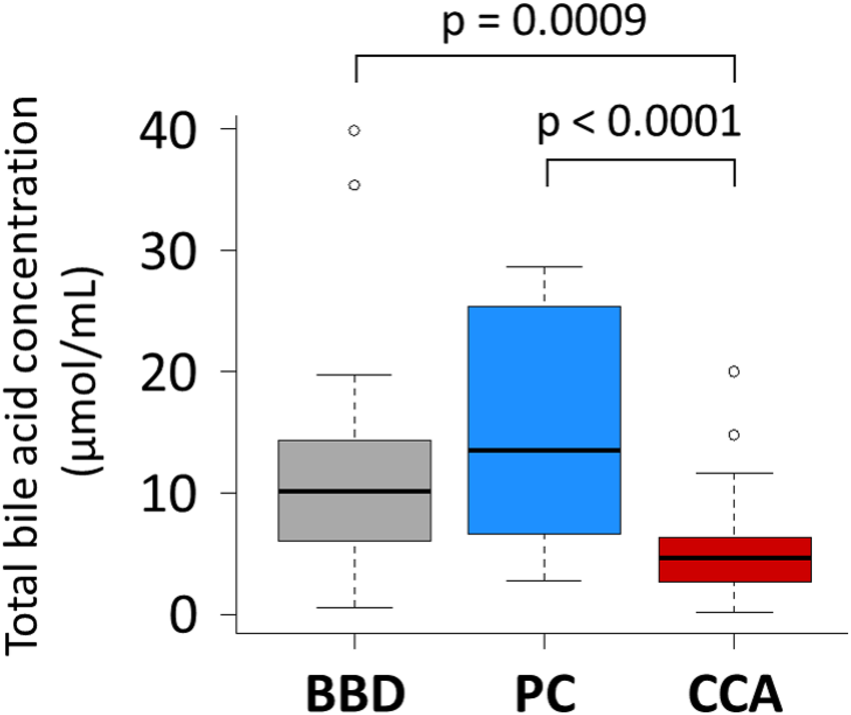
Comparison of total bile acid concentration in patients with benign biliary diseases, pancreatic cancer and cholangiocarcinoma.

Using the optimized LC-MS/MS method, we quantified the 15 major bile acids from the bile of BBD (n = 57), PC (n = 17) and CCA (n = 30) patients. The average concentration of bile acids was the highest (15.3 μmol/mL) in PC patients (Fig. 2). A recent study indicated that the increase in biliary bile acids increases the pancreaticobiliary reflux, which triggers acute and chronic pancreatitis, leading to PC^22^. In addition, the average concentration of total bile acids in the BBD group (10.7 μmol/mL) was twice as high as the CCA group (5.4 μmol/mL) (Supplementary Fig. S5), which was consistent with the results reported by Park *et al*.^16^. The low levels of biliary bile acid in CCA patients may result from decreased bile acid excretion from bile duct epithelium due to bile duct obstruction and inflammation^23^.

**Figure 3:**
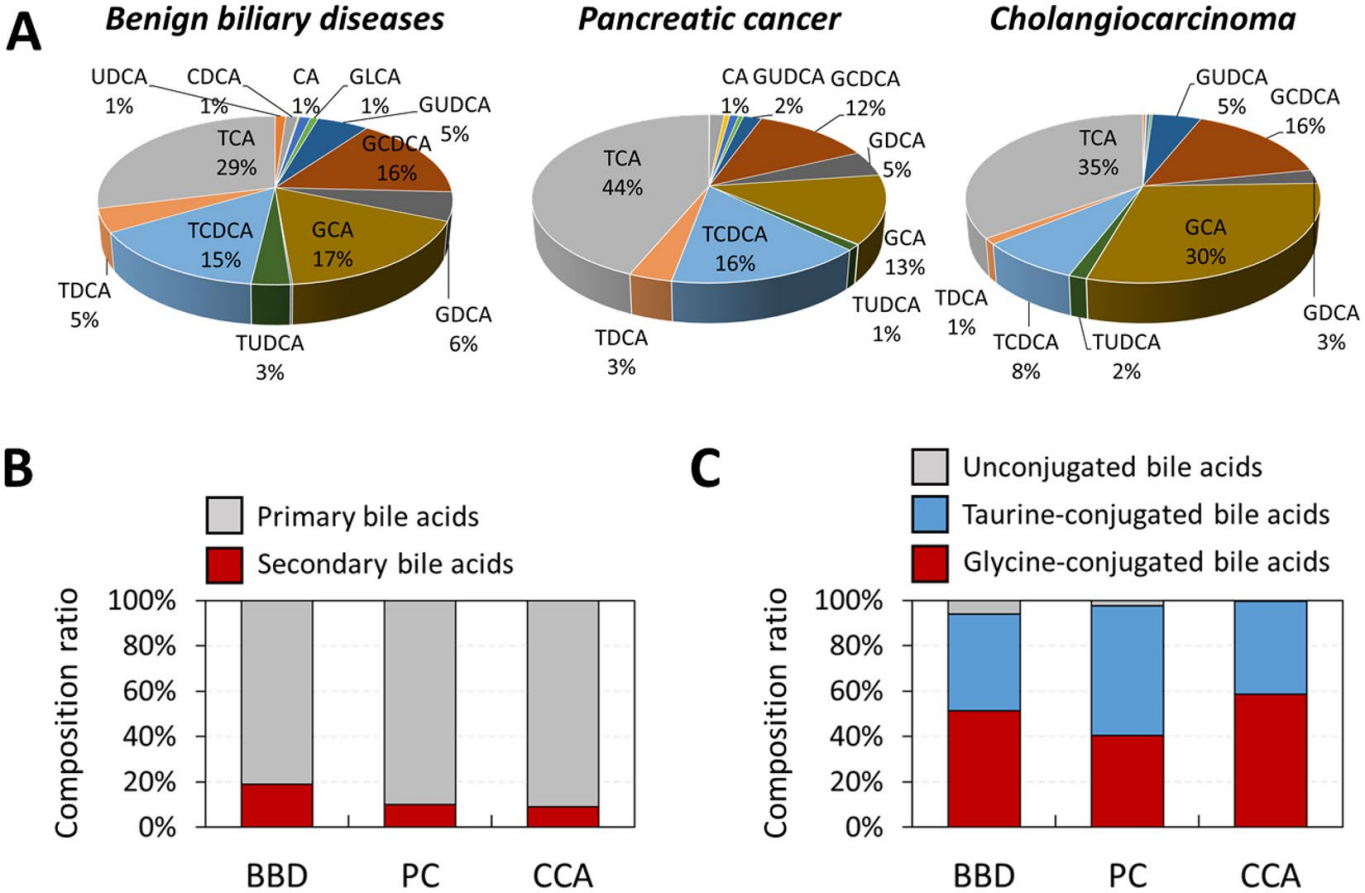
(**A**) Comparison of bile acid composition ratio of cholangiocarcinoma and benign biliary diseases. (**B**) Comparison of composition ratio of primary bile acids and secondary bile acids. (**C**) Comparison of composition ratio of unconjugated bile acids, taurine-conjugatd bile acids and glycine-conjugated bile acids.

The bile acids can be categorized according to the location of synthesis (primary and secondary) and conjugated molecules (glycine, taurine and un-conjugated)^24^. Our quantitative analysis showed that the average proportion of secondary bile acids was nearly twice that of patients with BBD compared with PC (p = 0.009) or CCA (p = 0.0006) (Fig. 3). The secondary bile acids are produced from primary bile acids by gut microbiota^25^. Recent studies have reported that dysbiosis of gut microbiota may induce hepatobiliary disease by changing the bile acid pool^26^. Secondary bile acids such as UDCA also protect cholangiocytes, suggesting that a reduction in secondary bile acid levels may result in biliary injury^27^.

The average proportion of total un-conjugated bile acids in CCA (0.6%) patients was 10 times lower than in BBD patients (6.0%, p = 0.03). Un-conjugated bile acids inhibit the growth of CCA cell lines^28^. Conversely, conjugated bile acids activate the epidermal growth factor receptor and increase the expression of cyclooxygenase-2, an endogenous enzyme present in many cancers^29^. In addition, they promote the growth of CCA cells and contribute to the activation of nuclear factor-kappa B, which blocks apoptosis^29^. They also promote growth and invasiveness of CCA via activation of S1PR2 rather than unconjugated bile acids^30^. Therefore, the increased proportion of conjugated bile acids in CCA patients is implicated in the pathogenesis of CCA.

### Discovery of CCA-associated bile acid biomarkers

**Figure 4:**
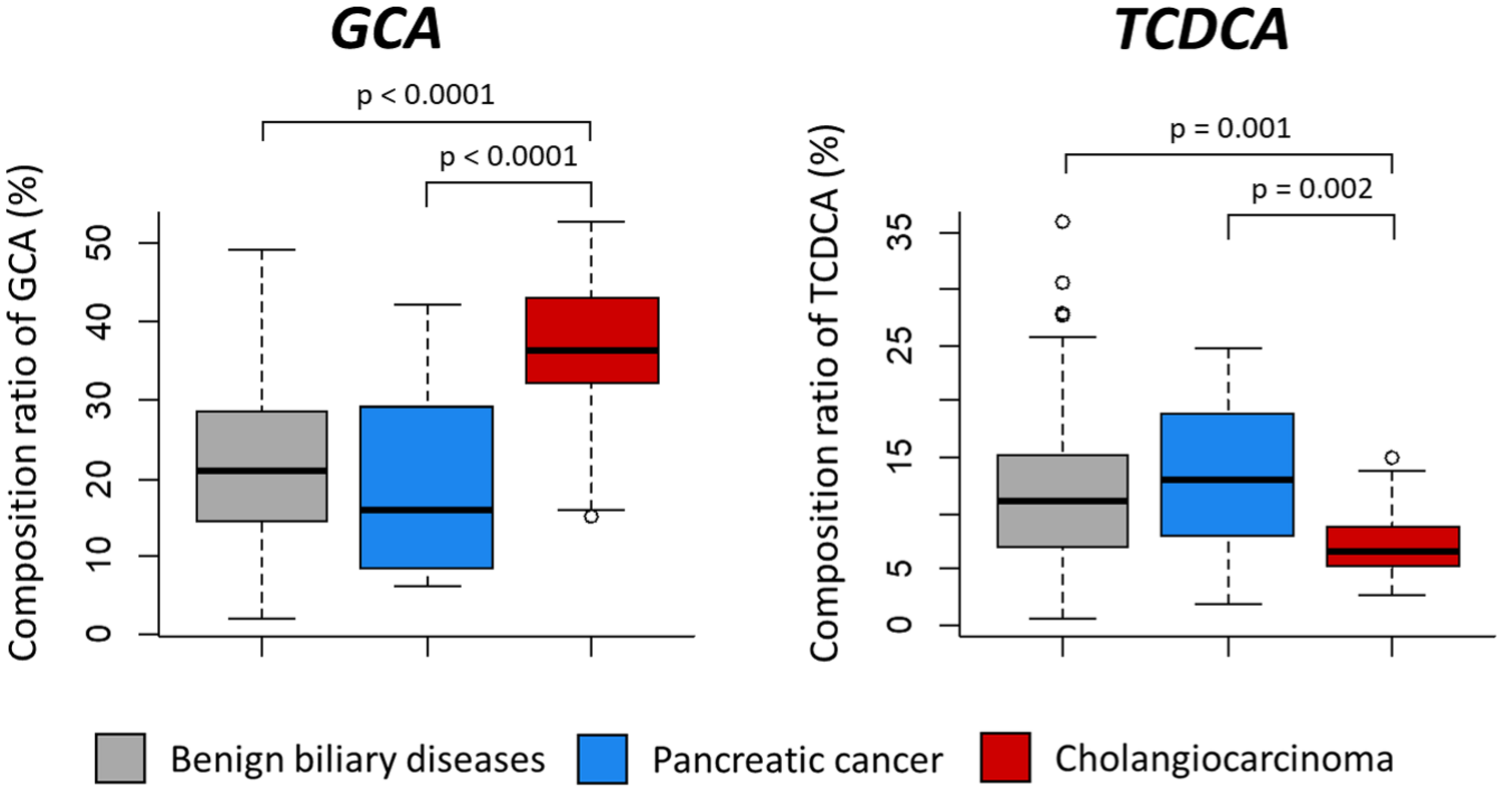
Comparison of GCA and TCDCA composition ratio in patients with benign biliary disease, pancreatic cancer and cholangiocarcinoma.

In this study, we identified a bile acid biomarker of CCA via quantitative analysis of 15 biliary bile acids in BBD, PC and CCA patients. Statistical significance of the average composition ratio of each biliary bile acid was analyzed by ANOVA (Analysis of variance) among CCA patients and other patient groups (Table 1). The average composition of GCA was the highest in CCA (35.6%) compared with BBD (22.3%, p < 0.0001) and PC (19.9%, p < 0.0001) (Fig. 4). In contrast to GCA, the average composition of TCDCA was significantly lower in CCA patients (7.31%) than BBD (12.45%, p = 0.001) and PC (13.82%, p = 0.002) patients. Therefore, we selected GCA as a positive biomarker of CCA and TCDCA as a negative biomarker.

**Table 1:** Comparison table of the average composition ratio (%) of 15 biliary bile acids in CCA, BBD and PC patients.

	**CCA**	**BBD (p-value)**		**PC (p-value)**	
LCA	0.04	0.02	(NS)	0.02	(NS)
UDCA	0.15	1.32	(NS)	0.02	(NS)
CDCA	0.10	1.67	(NS)	1.00	(NS)
DCA	0.00	0.27	(NS)	0.31	(NS)
CA	0.27	2.37	(NS)	1.13	(NS)
GLCA	0.13	0.72	(NS)	0.32	(NS)
GUDCA	5.44	6.03	(NS)	2.66	(NS)
GCDCA	16.06	17.10	(NS)	14.22	(NS)
GDCA	1.37	4.97	(0.0009)	3.39	(0.2)
GCA	35.58	22.33	(<0.0001)	19.95	(<0.0001)
TLCA	0.01	0.10	(0.006)	0.06	(0.3)
TUDCA	1.48	2.75	(NS)	1.00	(NS)
TCDCA	7.31	12.45	(0.001)	13.82	(0.002)
TDCA	0.48	2.74	(0.004)	2.14	(0.1)
TCA	31.56	25.15	(0.04)	39.96	(0.04)

Datasets were analyzed using one-way analysis of variance (ANOVA) to determine if there is a significant difference in the average composition ratio (%) of biliary bile acids in CCA, BBD and PC patients. NS indicates that the ANOVA did not show a significant difference (p < 0.05) among the three patient groups. When ANOVA p < 0.05, p-values are indicated for pairwise comparisons between CCA and BBD patients and between CCA and PC patients (protected Fisher’s least significant difference (LSD) posthoc test).

### Effects of bile acids on the expression of FXR, TGR5 and S1PR2 genes in CCA cell line

**Figure 5:**
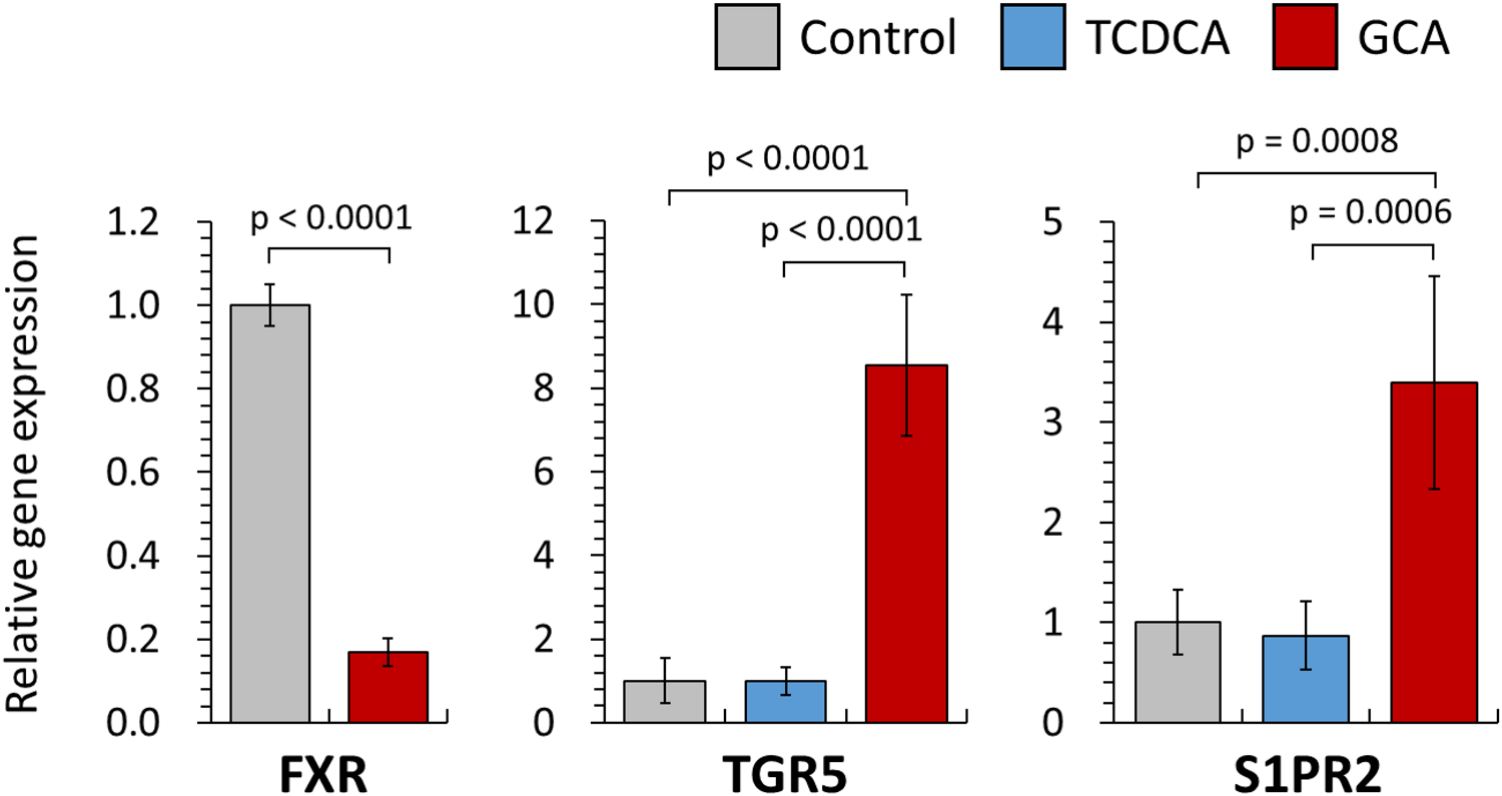
Comparison of relative gene expression of FXR, TGR5, and S1PR2 treating GCA and TCDCA in CCA cell lines.

Bile acids bind to several bile acid receptors in the cholangiocyte resulting in various biological phenotypes. In particular, quantitative changes in bile acid receptors such as FXR, TGR5, and S1PR2 affect the development and activity of CCA cells^29^. Therefore, we quantified the changes in gene expressions for FXR, TGR5, and S1PR2 by qRT-PCR to verify the phenotypical effects of GCA and TCDCA (Fig. 5). TCDCA was selected as a negative biomarker of CCA, and therefore, was expected to have reduced gene expression related to CCA pathogenesis compared with GCA. As control samples, no bile acids were treated with CCA cell line.

FXR is a bile acid receptor mainly present in liver and intestine, and mediates the pathogenesis of various biliary diseases as well as bile acid transport and metabolism^31^. Several studies have reported that a lower expression of FXR was linked to various biliary diseases due to abnormal bile acid transport^28^. In our transcriptomic analysis, the expression of FXR gene in GCA-treated (0.17-fold less versus control) cell lines was lower than in control. The decrease in FXR gene expression has been reported to occur human CCA, suggesting that GCA closely correlated with tumor differentiation^32^.

Interestingly, the TGR5 gene expression was 8.55 times higher in GCA-treated CCA cell lines than in control (p < 0.0001) or TCDCA-treated cells (p < 0.0001). TGR5 is a G-protein-coupled receptor present in cholangiocytes, activated in response to bile acids, and regulates the proliferation of cholangiocytes^33,34^. Thus, overexpression of TGR5 in CCA promotes cell proliferation, induces resistance to apoptosis, and contributes to tumor pathogenesis^35^. Therefore, we demonstrated that GCA may affect the development of CCA due to the elevated TGR5 expression in CCA cell line treated with GCA. In addition, the S1PR2 gene expression in GCA-treated CCA cell line was 3.4-fold higher compared with control (p = 0.0008) and 3.9-fold higher compared with TCDCA-treated cells (p = 0.0006), respectively. S1PR2 is also a G-protein-coupled receptor activated by sphingosine 1-phosphate, resulting in cell proliferation by ERK1/2 and protein kinases B in cholangiocytes^36^. Taken together, we propose GCA and TCDCA as phenotypic biomarkers that distinguish CCA from BBD and PC. Moreover, the metabolic profiling strategy integrated with the gene expression data is highly reliable and has the strongest diagnostic potential.

## Conclusion

In this study, we used LC-MS/MS to quantitatively analyze 15 biliary bile acids in patients diagnosed with CCA, BBD and PC to discover CCA-specific metabolic biomarkers. Because the average composition ratio of GCA and TCDCA in CCA patients varied significantly compared with BBD and PC, they were selected as positive and negative biomarkers of CCA, respectively. To validate the biological effects of GCA and TCDCA on CCA, our analysis further revealed that the gene expression levels of TGR5 and S1PR2 were remarkably enhanced in CCA cells treated with GCA, compared with control and TCDCA. Therefore, we concluded that GCA can be a promising metabolic biomarker for the specific detection of CCA and that TCDCA represented a negative marker. We will use the selected biomarkers in this study, GCA and TCDCA, to validate the accuracy of biomarkers in larger number of patient samples and perform additional mechanistic studies by *in vivo* experiments.

## Materials and Methods

### Materials and reagents

Lithocholic acid (LCA), ursodeoxycholic acid (UDCA), chenodeoxycholic acid (CDCA), DCA, cholic acid (CA), GCA, taurodeoxycholic acid (TDCA), and taurocholic acid (TCA) were purchased from Sigma Aldrich (MO, USA). Glycolithocholic acid (GLCA), tauroursodeoxycholic acid (TUDCA) and taurochenodeoxycholic acid (TCDCA) were obtained from Carbosynth (Berkshire, UK) and glycochenodeoxycholic acid (GCDCA), glycodeoxycholic acid (GDCA) and taurolithocholic acid (TLCA) were purchased from Santa Cruz (TX, USA). Glycoursodeoxycholic acid (GUDCA) was obtained from Achemblock (CA, USA). HPLC-grade water, methanol and acetonitrile are products of Duksan (Ansan, Korea).

### Patient and bile collection

Bile samples were collected from Samsung Medical Center (Seoul, Korea). The study protocol was reviewed and approved by the Institutional Review Board of the Samsung Medical Center (IRB No. 2013-02-025). All experiments and methods were performed in accordance with the relevant guidelines and regulations. Written informed consent was obtained from all participants. The samples were obtained from 57 patients with BBD, 17 patients with PC, and 30 patients with CCA. These bile samples were stored at −70 °C until the preparation of bile acids.

### Bile acid preparation

After thawing 30 μL of bile samples on ice, the bile acids were extracted with 400 μL of chloroform and 200 μL of methanol. After mixing well, 120 μL of distilled water was added to the mixture. The mixture was vortexed and centrifuged for 5 min, followed by transfer of 100 μL of the supernatant to a 1.5 mL micro tube, and drying in a centrifugal vacuum concentrator. After complete drying, bile acids were dissolved in 100 μL of 50% methanol prior to transfer to HPLC autosampler vials for LC-MS/MS analysis.

### LC-MS/MS analysis

Re-suspended bile acids (10 μL) were injected onto Unison UK-C18 column (3 µm, ID 3 mm × 100 mm). LC-MS/MS analysis was performed using an integrated system composed of Accela LC (ThermoFisher, MA, USA) and TSQ Quantum access max (ThermoFisher, MA, USA). Bile acids were separated on the analytical column at a flow rate of 400 μL/min. The LC gradient method was set as follows: t = 0 min, 35% B; t = 2 min, 35% B; t = 20 min, 50% B; t = 30 min, 80% B; t = 33 min, 80% B; t = 35 min, 35% B; t = 40 min 35% B. Solvent A comprised 100% water and 0.1% (v/v) formic acid, and solvent B was 100% acetonitrile and 0.1% (v/v) formic acid. The mass spectrometer was operated in negative ion mode. An electrospray ionization spray voltage was used at 3 kV, and the capillary temperature was 300 °C.

### Cell culture

CCA cell lines, designated SNU-245, were purchased from Korean Cell Line Bank^37^. The cells were cultured in RPMI-1640 medium (Biowest, Nuaillé, France) supplemented with 10% fetal bovine serum (Biowest, Nuaillé, France) and 1 mg/mL gentamicin (Sigma Aldrich, MO, USA) for 5 days in a humidified incubator at 37 °C in an atmosphere of 5% CO_2_. GCA and TCDCA were dissolved in 100 μL of DMSO (Sigma Aldrich, MO, USA), respectively, and added to the medium to obtain a final concentration of 1.6 μmol/mL. For control, 100 μL of DMSO was added to the media without any bile acids. Cells were cultured for 48 h after treatment with each bile acid.

### RNA preparation and qRT-PCR

The cells were removed from the adherent surface using trypsin (Biowest, Nuaillé, France). The total RNA was isolated from the collected cells using QIAGEN RNA mini kit (QIAGEN, Hilden, Germany). The RNA concentration and purity were confirmed by spectrophotometry (ThermoFisher, MA, USA) and gel electrophoresis, respectively. Reverse transcription was performed with cDNA using M-MLV reverse transcriptase (Promega, WI, USA) and oligo (dT) primer (ThermoFisher, MA, USA). PCR primer sequences of FXR, S1PR2, TGR5 and *β*-actin were reported previously^28,30,38^, and *β*-actin was used as an internal control for RT-PCR. The RT-PCR assay was performed using the LightCycler 480 II (Roche, Basel, Swiss) under the following conditions: 45 cycles of denaturation at 95 °C for 20 sec, annealing at 60 °C for 20 sec, and extension at 72 °C for 20 sec.

### Statistical analysis

Boxplots were created in R by using the boxplot() function. Analysis of variance (ANOVA) and Fisher’s least significant difference (LSD) tests were conducted using GraphPad Prism version 7.0 c.

## Electronic supplementary material

Supplementary Information
