# Supplementary material for: Discovery of glycocholic acid and taurochenodeoxycholic acid as phenotypic biomarkers in cholangiocarcinoma
Source: Sci Rep. 2018 Jul 23;8:11088. doi: 10.1038/s41598-018-29445-z (PMC6056462; doi:10.1038/s41598-018-29445-z)
Supplement: Supplementary file 1 — Supplementary Information [file 41598_2018_29445_MOESM1_ESM.docx]

**Discovery of glycocholic acid and taurochenodeoxycholic acid as phenotypic biomarkers in cholangiocarcinoma**

Won-Suk Song^1,+^ , Hae-Min Park^2,+^, Jung Min Ha^3^, Sung Gyu Shin^4^, Han-Gyu Park^4^, Da-Hee Ahn^4^, Sung-Min Kim^4^, Joonwon Kim^1^, Yung-Hun Yang^5^, Tianzi Zhang^6^, Jae Hyun Jeong^4^, Ashleigh B. Theberge^6^, Byung-Gee Kim^1^, Jong Kyun Lee^3,^* and Yun-Gon Kim^4,^*

*^1^School of Chemical and Biological Engineering, Seoul National University, Seoul 08826, Korea*

*^2^Departments of Chemistry and Molecular Biosciences, Northwestern University, Evanston, Illinois 60208, United States*

*^3^Division of Gastroenterology, Department of Medicine, Samsung Medical Center, Sungkyunkwan University, Seoul 06351, Korea*

*^4^Department of Chemical Engineering, Soongsil University, Seoul 06978, Korea*

*^5^Department of Biological Engineering, Konkuk University, Seoul 05029, Korea*

*^6^Department of Chemistry, University of Washington, Box 351700, Seattle, WA 98195, United States*

^*^Corresponding author contact information:

Yun-Gon Kim, Ph.D., Professor

Department of Chemical Engineering, Soongsil University

369 Sangdo-Ro, Seoul, Korea

E-mail: [ygkim@ssu.ac.kr](mailto:ygkim@ssu.ac.kr)

Phone: +82-2-828-7099

Jong-Kyun Lee, M.D., Ph.D. Professor

Devision of Gastroenterology,

Department of Medicine Samsung Medical Cencer,

Sungkyunkwan University School of Medicine

81 Irwon-Ro, Seoul, Korea

E-mail: [jongk.lee@samsung.com](mailto:jongk.lee@samsung.com)

Phone: +82-2-828-7099

^+^These authors contributed equally to this work

**Supplementary information figures**

**Supplementary figure S1.** 15 species of human major bile acids.


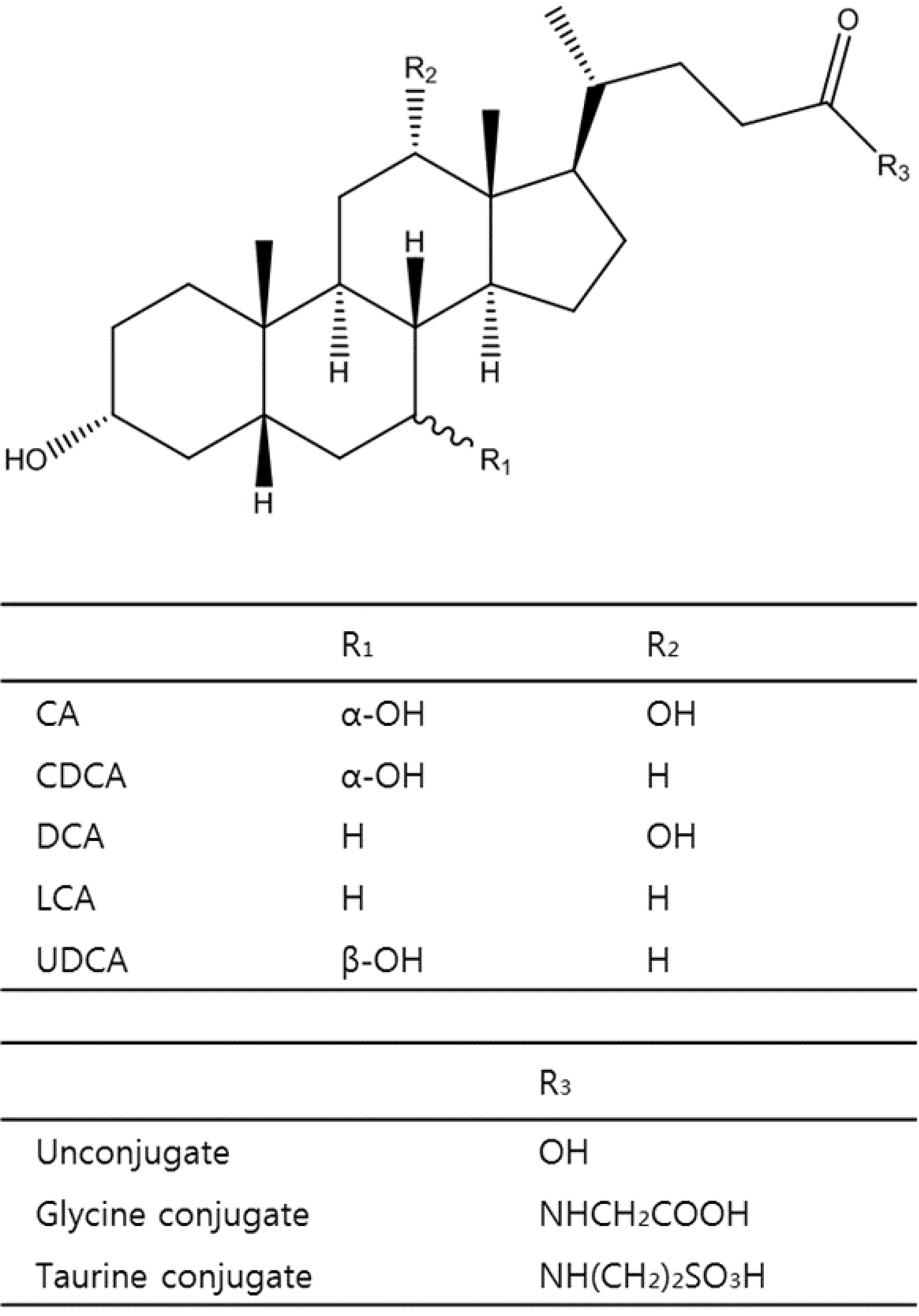


**Supplementary figure S2.** Comparison of full mass spectra with unconjugated bile acids and glycine-, taurine-conjugated bile acids.

**
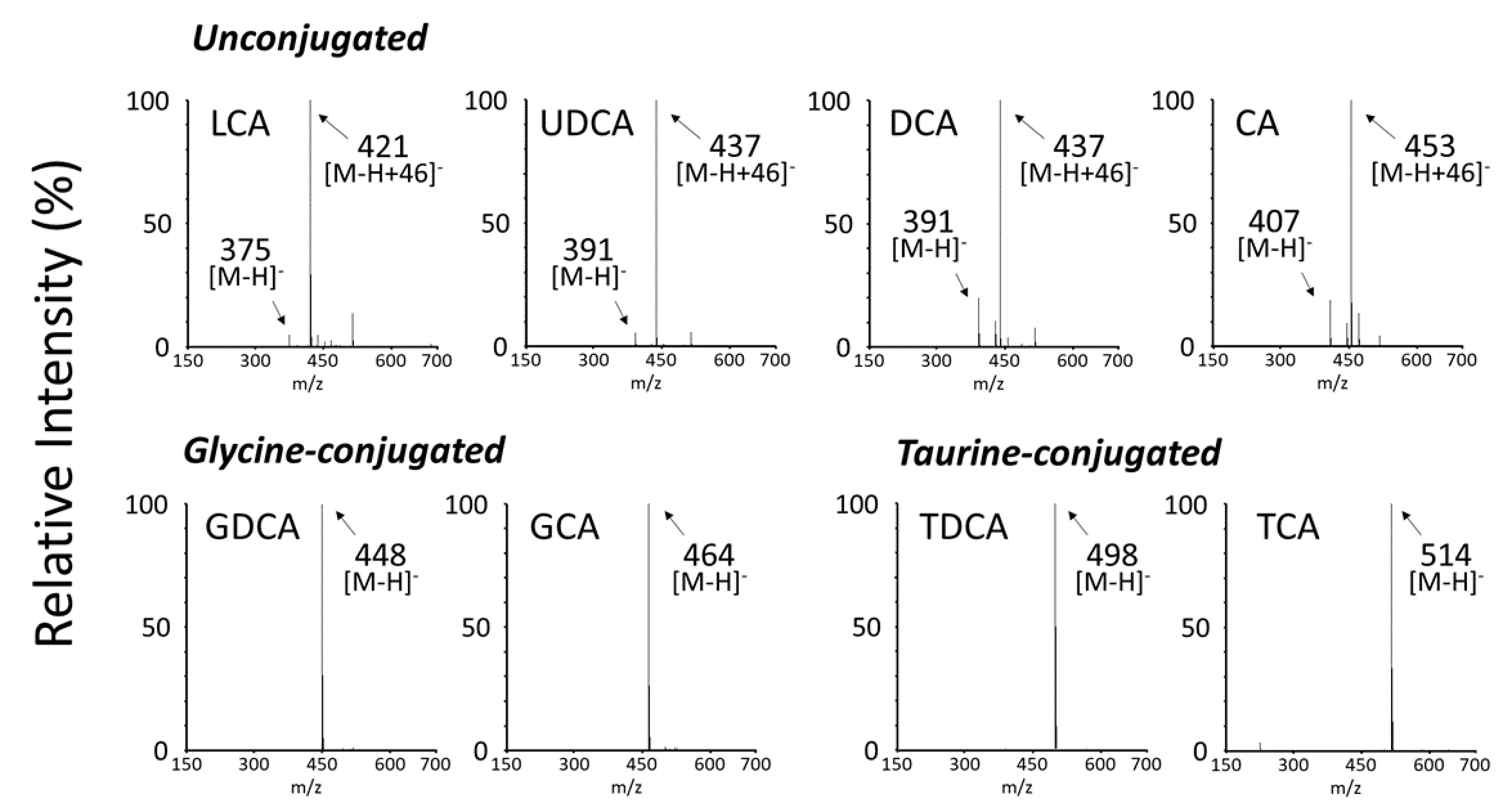
**

**Supplementary figure S3.** The fragment ion mass spectrum of unconjugated bile acid (UDCA), glycine-conjugated bile acid (GDCA) and taurine-conjugated bile acid (TDCA).

**
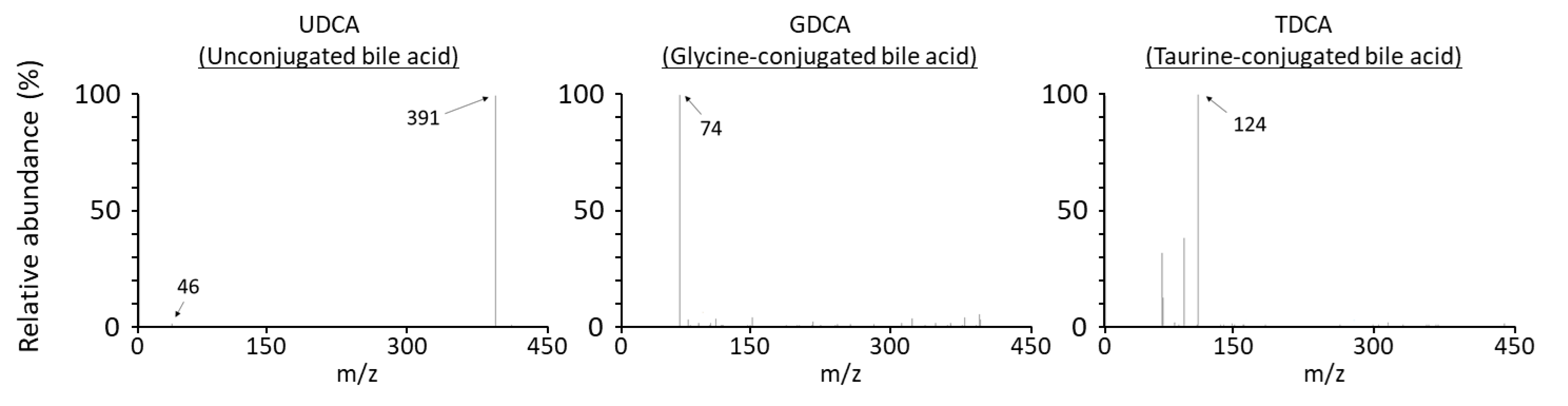
**

**Supplementary figure S4.** Comparison of benign biliary diseases, pancreatic cancer and cholangiocarcinoma with TIC and SRM chromatograms.

**
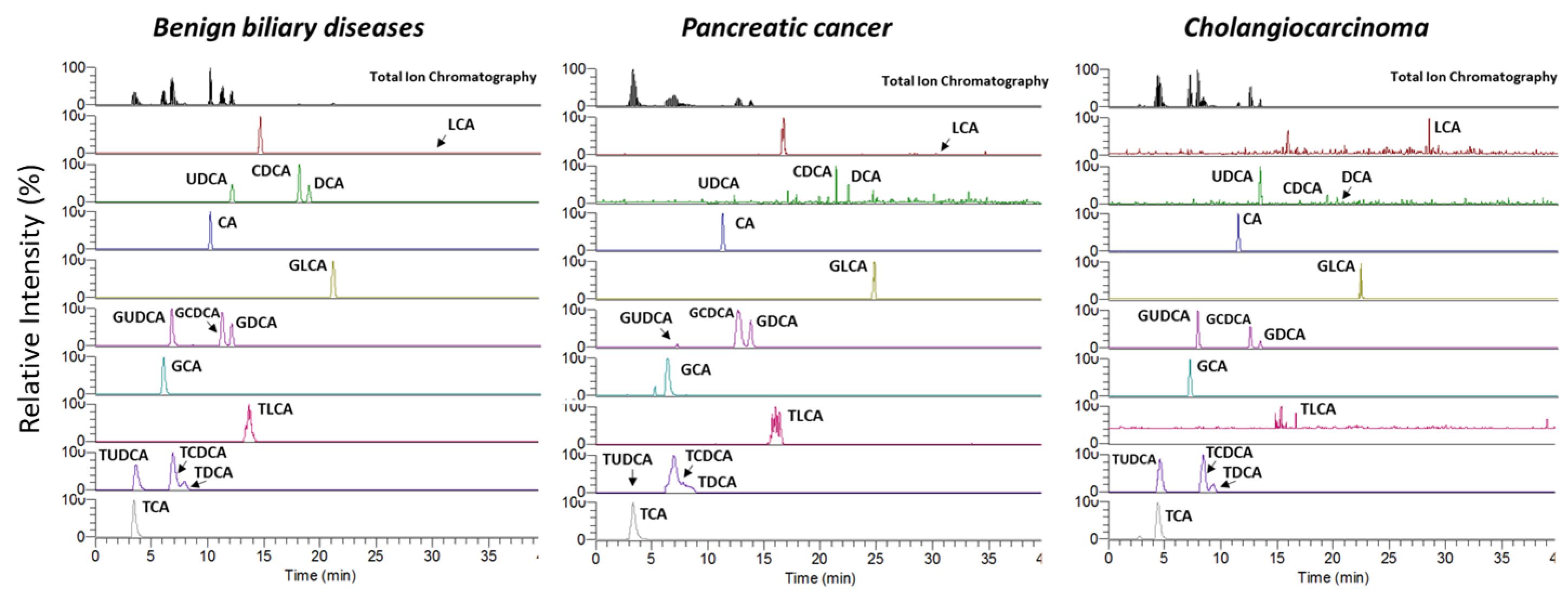
**

**Supplementary figure S5.** Box plots comparing each bile acid concentration in patients with BBD, PC, and CCA.

**
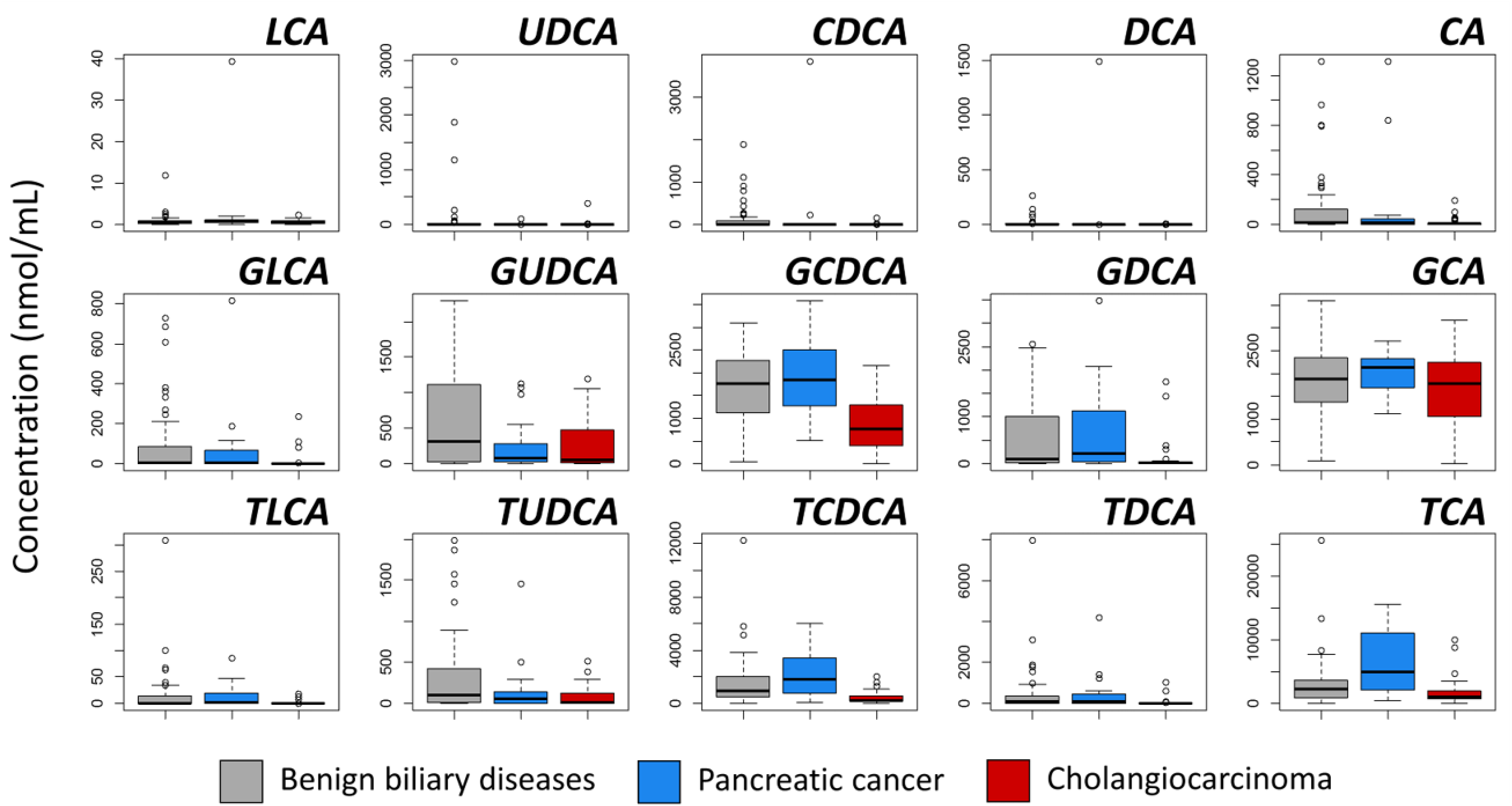
**
